# A Classical Interpretation of the Scrooge Distribution

**DOI:** 10.3390/e20080619

**Published:** 2018-08-20

**Authors:** William K. Wootters

**Affiliations:** Department of Physics, Williams College, Williamstown, MA 01267, USA; william.wootters@williams.edu; Tel.: +1-413-597-2156

**Keywords:** subentropy, GAP measure, accessible information

## Abstract

The Scrooge distribution is a probability distribution over the set of pure states of a quantum system. Specifically, it is the distribution that, upon measurement, gives up the *least* information about the identity of the pure state compared with all other distributions that have the same density matrix. The Scrooge distribution has normally been regarded as a purely quantum mechanical concept with no natural classical interpretation. In this paper, we offer a classical interpretation of the Scrooge distribution viewed as a probability distribution over the probability simplex. We begin by considering a real-amplitude version of the Scrooge distribution for which we find that there is a non-trivial but natural classical interpretation. The transition to the complex-amplitude case requires a step that is not particularly natural but that may shed light on the relation between quantum mechanics and classical probability theory.

## 1. Introduction

In the early days of quantum information theory, the term “quantum communication” would typically have been understood to refer to the transmission of *classical* information via quantum mechanical signals. Such communication can be done in a sophisticated way, with the receiver making joint measurements on several successive signal particles [1,2], or it can be done in a relatively straightforward way with the receiver performing a separate measurement on each individual signal particle. In both cases, but especially in the latter case, a particularly interesting quantity, given an ensemble of quantum states to be used as an alphabet, is the ensemble’s *accessible information*. This is the maximum amount of information that one can obtain about the identity of the state, on average, by making a measurement on the system described by the specified ensemble. The average here is over the outcomes of the measurement, and the maximization is over all possible measurements. In general, accessible information can be defined for ensembles consisting of pure and mixed states, but in this paper, we consider only pure-state ensembles.

Any ensemble $\left\{(|\psi_{j}\rangle ,p_{j})\right\}$ of pure quantum states with their probabilities has a unique density matrix. However, for any given density matrix ρ representing more than a single pure state, there are infinitely many ensembles—“ρ-ensembles”—described by that density matrix. Thus, it is natural to ask the following question: for a given density matrix ρ, what pure-state ρ-ensemble has the greatest value of the accessible information and what pure-state ρ-ensemble has the lowest value? The former question was answered by an early (1973) result in quantum information theory [3]—the pure-state ρ-ensemble with the greatest accessible information is the one consisting of the eigenstates of ρ with weights given by the eigenvalues. The latter question was answered in a 1994 paper [4], in which the ρ-ensemble minimizing the accessible information was called the Scrooge ensemble, or Scrooge distribution, since it is the ensemble that is most stingy with its information.

To see a simple example, consider a spin-1/2 particle whose density matrix ρ has the ∣↑〉 and ∣↓〉 states as its eigenvectors, with eigenvalues $\lambda_{↑}$ and $\lambda_{↓}$. The eigenstate ensemble for ρ, that is, the ρ-ensemble from which one can extract the most information, is the two-state ensemble consisting of the ∣↑〉 state with probability $\lambda_{↑}$ and the ∣↓〉 state with probability $\lambda_{↓}$. The optimal measurement in this case—the measurement that provides the most information—is the up-down measurement, and the amount of information it provides is equal to the von Neumann entropy of the density matrix:

(1) $$I=S\left(\rho \right)=-\left(\lambda_{↑}\ln\lambda_{↑}+\lambda_{↓}\ln\lambda_{↓}\right).$$

On the other hand, the Scrooge ensemble for this density matrix is represented by a continuous probability distribution over the whole surface of the Bloch sphere. If $\lambda_{↑}$ is larger than $\lambda_{↓}$, then this continuous distribution is weighted more heavily towards the top of the sphere. We can write the Scrooge distribution explicitly in terms of the variable x=(1+cosθ)/2, where θ is the angle measured from the north pole:

(2) $$\sigma \left(x\right)=\frac{2}{\lambda_{↑}\lambda_{↓}}\cdot \frac{1}{{\left(\frac{x}{\lambda_{↑}}+\frac{1-x}{\lambda_{↓}}\right)}^{3}}.$$

The probability density σ(x) is normalized in the sense that $\int_{0}^{1}\sigma \left(x\right)dx=1$ (the distribution is uniform over the azimuthal angle). Again, this is the ensemble of pure states from which one can extract the least information about the identity of the pure state, among all ensembles with the density matrix ρ. Somewhat remarkably, the average amount of information one gains by measuring this particular ensemble is entirely independent of the choice of measurement, as long as the measurement is complete—that is, as long as each outcome is associated with a definite pure state. This amount of information comes out to be a quantity called the subentropy *Q* of the density matrix:

(3) $$I=Q\left(\rho \right)=-\frac{\lambda_{↑}^{2}\ln\lambda_{↑}-\lambda_{↓}^{2}\ln\lambda_{↓}}{\lambda_{↑}-\lambda_{↓}}.$$

We give more general expressions for both the Scrooge ensemble and the subentropy in Section 2 below.

In recent years, the Scrooge distribution has made other appearances in the physics literature. Of particular interest is the fact that this distribution has emerged from an entirely different line of investigation, in which the system under consideration is entangled with a large environment and the whole system is in a pure state. In that case, if one looks at the *conditional* pure states of the original system relative to the elements of an orthogonal basis of the environment, one typically finds that these conditional states are distributed by a Scrooge distribution [5,6,7,8]. In this context, the distribution is usually called a GAP measure (Gaussian adjusted projected measure, the three adjectives corresponding to the three steps by which the measure can be constructed). On another front, the Scrooge distribution has been used to address the difficult problem of bounding the *locally* accessible information when there is more than one receiver [9].

Meanwhile, the concept of subentropy, which originally arose (though without a name) in connection with the outcome entropy of random measurements [10,11], has appeared not only in problems concerning the acquisition of classical information [12,13,14], but also in the quantification of entanglement [15] and the study of quantum coherence [16,17,18,19]. Many detailed properties of subentropy have now been worked out, especially concerning its relation to the Shannon entropy [20,21,22,23,24].

Though it is possible to devise a strictly classical situation in which subentropy arises [22], the Scrooge distribution has generally been regarded as a purely quantum mechanical concept. It is, after all, a probability distribution over pure quantum states. The aim of this paper is to provide a classical interpretation of the Scrooge distribution, and in this way, to provide a new window into the relation between quantum mechanics and classical probability theory.

We find that it is much easier to make the connection if we begin by considering not the standard Scrooge distribution, but rather the analogous distribution one obtains for the case of quantum theory with *real* amplitudes. In that case, the dimension of the set of pure states is the same as the dimension of the associated probability simplex, and we find that there is a fairly natural distribution within classical probability theory that is essentially identical to the real-amplitude version of the Scrooge distribution. This distribution arises as the solution to a certain classical communication problem that we describe in Section 4.

With this interpretation of the real-amplitude Scrooge distribution in hand, we ask how the classical communication scenario might be modified to arrive at the original Scrooge ensemble for standard, complex-amplitude quantum theory. As we will see, the necessary modification is not particularly natural, but it is simple.

Thus, we begin in Section 2 and Section 3 by reviewing the derivation of the Scrooge distribution and by working out the analogous distribution for the case of real amplitudes. Then, in Section 4, we set up and analyze the classical communication problem that, as we show in Section 5, gives rise to a distribution that is equivalent to the real-amplitude Scrooge distribution. In Section 6, we modify the classical communication scenario to produce the standard, complex-amplitude Scrooge distribution. Finally, we summarize and discuss our results in Section 7.

## 2. The Scrooge Distribution

There are several ways in which one can generate the Scrooge distribution. In this section, we review the main steps of the derivation given in Ref. [4], which applies to a Hilbert space of finite dimension. (The distribution can also be defined for an infinite-dimensional Hilbert space [5,6,7,8].) We begin by setting up the problem.

We imagine the following scenario. One participant, Alice, prepares a quantum system with an *n*-dimensional Hilbert space in a pure state |x〉 and sends it to Bob. Bob then tries to gain information about the identity of this pure state. Initially, Bob’s state of knowledge is represented by a probability density σ(x) over the set of pure states. (The symbol *x* represents a multi-dimensional parameterization of the set of pure states.) Bob makes a measurement on the system and thereby gains information. The amount of information he gains may depend on the outcome he obtains, so we are interested in the *average* amount of information he gains about *x*, the average being over all outcomes.

The standard quantification of Bob’s average gain in information is the Shannon mutual information between the identity of the pure state and the outcome of the measurement. We can express this mutual information in terms of two probability functions: (i) the probability p(j|x) of the outcome *j* when the state is |x〉, and (ii) the overall probability p(j)=∫σ(x)p(j|x)dx of the outcome *j* averaged over the whole ensemble. In terms of these functions, the mutual information is

(4) $$I=-\sum_{j}p\left(j\right)\ln p\left(j\right)+\int \sigma \left(x\right)[\sum_{j}p\left(j|x\right)\ln p\left(j|x\right)]dx.$$

The *accessible information* of the ensemble defined by σ(x) is the maximum value of the mutual information *I*, where the maximum is taken over all possible measurements.

Again, for a given density matrix ρ, the Scrooge distribution is defined to be the pure-state ρ-ensemble with the lowest value of the accessible information. One can obtain the Scrooge distribution via the following algorithm [4].

We start by recalling the concept of “ρ distortion.” Consider for now a finite ensemble $\left\{(|\psi_{i}\rangle ,p_{i})\right\}$ of pure states (i=1,…,m) whose density matrix is the completely mixed state:

(5) $$\sum_{i=1}^{m}p_{i}|\psi_{i}\rangle \langle \psi_{i}|=\frac{1}{n}I.$$

Let $|{\tilde{\psi }}_{i}\rangle$ be the subnormalized state vector $|{\tilde{\psi }}_{i}\rangle =\sqrt{p_{i}}|\psi_{i}\rangle$, so that

(6) $$\sum_{i=1}^{m}|{\tilde{\psi }}_{i}\rangle \langle {\tilde{\psi }}_{i}|=\frac{1}{n}I.$$

Under ρ distortion, each vector $|\tilde{\psi }\rangle$ is mapped to another subnormalized vector $|\tilde{\phi }\rangle$ defined by

(7) $$|{\tilde{\phi }}_{i}\rangle =\sqrt{n\rho }|{\tilde{\psi }}_{i}\rangle .$$

Note that the density matrix formed by the $|{\tilde{\phi }}_{i}\rangle$’s is ρ:

(8) $$\sum_{i=1}^{m}|{\tilde{\phi }}_{i}\rangle \langle {\tilde{\phi }}_{i}|=\sqrt{n\rho }\left(\frac{1}{n}I\right)\sqrt{n\rho }=\rho .$$

In terms of normalized vectors, the new ensemble is $\left\{(|\phi_{i}\rangle ,q_{i})\right\}$, with the new probabilities $q_{i}$ equal to

(9) $$q_{i}=\langle {\tilde{\phi }}_{i}|{\tilde{\phi }}_{i}\rangle =np_{i}\langle \psi_{i}\left|\rho \right|\psi_{i}\rangle .$$

In this way, any ensemble having the completely mixed density matrix can be mapped to a “ρ distorted” ensemble with a density matrix ρ.

The Scrooge ensemble is a continuous ensemble, not a discrete one, but the concept of ρ distortion can be immediately extended to the continuous case, and the Scrooge distribution can be easily characterized in those terms; it is the ρ distortion of the *uniform* distribution over the unit sphere in Hilbert space. The uniform distribution is the unique probability distribution over the set of pure states that is invariant under all unitary transformations.

Let us see how the ρ distortion works out in this case. First, for the uniform distribution, it is convenient to label the parameters of the pure states by *y* instead of *x*, so that we can reserve *x* for the Scrooge distribution. Let τ(y) be the probability density over *y* that represents the uniform distribution over the unit sphere (a particular parameterization will be specified shortly). In terms of normalized states, a ρ distortion maps each pure state |y〉 into the pure state |x〉 defined by

(10) $$|x\rangle =\frac{\sqrt{\rho }|y\rangle }{\sqrt{\langle y|\rho |y\rangle }}.$$

This mapping defines *x* as a function of *y*: x=f(y). (We write *f* explicitly below.) The resulting probability density over *x* is obtained from the continuous version of Equation (9).

(11) σ(x)=nτ(y)〈y|ρ|y〉J(y/x).

Here, J(y/x) is the Jacobian of the *y* variables with respect to the *x* variables. On the right-hand side of Equation (11), each *y* is interpreted as $f^{-1}\left(x\right)$, so that we get an expression that depends only on *x*.

To get an explicit expression for the Scrooge distribution—that is, an explicit expression for the probability density σ(x)—we need to choose a specific set of parameters labeling the pure states. We choose the same set of parameters to label both the uniform distribution (where we call the parameters *y*) and the Scrooge distribution (where we call the parameters *x*). We define our parameters relative to a set of normalized eigenstates $|e_{j}\rangle$ of the density matrix ρ. A general pure state |x〉 can be written as

(12) $$|x\rangle =\sum_{j=1}^{n}a_{j}e^{-i\theta_{j}}|e_{j}\rangle ,$$

where each $a_{j}$ is a non-negative real number, and each phase $\theta_{j}$ runs from zero to 2π. For definiteness, employing the freedom to choose an overall phase, we define $\theta_{n}$ to be zero. We take *x* (or *y*) to consist of the following parameters: the squared amplitudes $x_{j}=a_{j}^{2}$ for j=1,…,n-1, and the phases $\theta_{j}$ for j=1,…,n-1. This set of 2n-2 parameters uniquely identifies any pure state. Later, we also use the symbol $x_{n}=1-x_{1}-\cdots -x_{n-1}$. Note that the $x_{j}$s are the probabilities of the outcomes of a particular orthogonal measurement associated with the eigenstates of ρ.

In terms of these parameters, the uniform distribution over the unit sphere takes a particularly simple form: it is the product of a uniform distribution over the phases and a uniform distribution over the (n-1)-dimensional probability simplex whose points are labeled by $\left\{x_{1},\ldots ,x_{n-1}\right\}$ [25]. The Scrooge distribution will likewise be a product and will be uniform over the phases but will typically have a certain bias over the probability simplex. Because the phases are always independent and uniformly distributed in the cases we consider, we omit the phases in our distribution expressions, writing the probability densities as functions of $\left\{x_{1},\ldots ,x_{n-1}\right\}$ (or $\left\{y_{1},\ldots ,y_{n-1}\right\}$).

Our aim now is to find explicit expressions for each of the factors appearing on the right-hand side of Equation (11). Since the uniform distribution over the unit sphere induces a uniform distribution over the probability simplex, the corresponding probability density τ(y) is a constant function, with the value of the constant being (n-1)! as required by normalization:

(13) $$\left(n-1\right)!\int_{0}^{1}\int_{0}^{1-y_{1}}\cdots \int_{0}^{1-y_{1}-\cdots -y_{n-2}}dy_{n-1}\cdots dy_{2}dy_{1}=1.$$

The function f(y) defined by the ρ-distortion map, Equation (10), is given by

(14) $$x_{j}=\frac{\lambda_{j}y_{j}}{\lambda_{1}y_{1}+\cdots +\lambda_{n}y_{n}},\;j=1,\ldots ,n-1,$$

where the $\lambda_{j}$’s are the eigenvalues of the density matrix ρ. One finds that the inverse map is

(15) $$y_{j}=\frac{x_{j}/\lambda_{j}}{x_{1}/\lambda_{1}+\cdots +x_{n}/\lambda_{n}},$$

and the Jacobian is

(16) $$J\left(y/x\right)=\frac{1}{\lambda_{1}\cdots \lambda_{n}}\cdot \frac{1}{{\left(\frac{x_{1}}{\lambda_{1}}+\cdots +\frac{x_{n}}{\lambda_{n}}\right)}^{n}}.$$

Meanwhile, the factor 〈y|ρ|y〉 can be written as

(17) $$\langle y|\rho |y\rangle =\lambda_{1}y_{1}+\cdots +\lambda_{n}y_{n}=\frac{1}{\frac{x_{1}}{\lambda_{1}}+\cdots +\frac{x_{n}}{\lambda_{n}}}.$$

By substituting the expressions from Equations (16) and (17) into Equation (11), we finally arrive at the probability density defining the Scrooge distribution:

(18) $$\sigma \left(x\right)=\frac{n!}{\lambda_{1}\cdots \lambda_{n}}\cdot \frac{1}{{\left(\frac{x_{1}}{\lambda_{1}}+\cdots +\frac{x_{n}}{\lambda_{n}}\right)}^{n+1}}.$$

This probability density is normalized in the sense that the integral over the probability simplex is unity:

(19) $$\int_{0}^{1}\int_{0}^{1-x_{1}}\cdots \int_{0}^{1-x_{1}-\cdots -x_{n-2}}\sigma \left(x\right)dx_{n-1}\cdots dx_{2}dx_{1}=1.$$

Now, how do we know that the distribution given by Equation (18) minimizes the amount of accessible information? First, one can show that for this distribution the mutual information *I* is independent of the choice of measurement as long as the measurement is complete [4]. So, one can compute the value of the accessible information by considering any such measurement, and the easiest one to consider is the orthogonal measurement along the eigenstates. The result is

(20) $$\mathrm{accessible}\;\mathrm{information}=-\sum_{k=1}^{n}\left(\prod_{l\neq k}\frac{\lambda_{k}}{\lambda_{k}-\lambda_{l}}\right)\lambda_{k}\ln\lambda_{k},$$

which defines the subentropy *Q*. One can also show that for *any* ρ-ensemble, the *average* mutual information over all complete orthogonal measurements is equal to *Q*, which implies that *Q* is always a lower bound on the accessible information. Since the Scrooge distribution *achieves* the value *Q*, it achieves the minimum possible accessible information among all ρ-ensembles.

## 3. The Real-Amplitude Analog of the Scrooge Distribution

Though our own world is described by standard quantum theory with complex amplitudes, we can also consider an analogous, hypothetical theory with real amplitudes. A pure state in the real-amplitude theory is represented by a real unit vector, and a density matrix is represented by a symmetric real matrix with non-negative eigenvalues and unit trace. Time evolution in this theory is generated by an antisymmetric real operator in place of the antihermitian operator iH.

The question considered in the preceding section can also be asked in regard to the real-amplitude theory. Given a density matrix ρ, we ask what ρ-ensemble has the smallest value of accessible information. It turns out that essentially all of the methods used in the preceding section continue to work in the real case. Again one begins with the uniform distribution over the unit sphere of pure states, and again, one obtains the Scrooge ensemble (in this case the real-amplitude Scrooge ensemble) via ρ distortion. The arguments leading to the conclusion that the ensemble produced in this way minimizes the accessible information work just as well in the real-amplitude case as in the complex-amplitude case.

The one essential difference between the two cases lies in the form of the initial probability density τ(y) that is associated with the uniform distribution over the unit sphere in Hilbert space. Whereas in the complex case the induced distribution over the probability simplex is uniform, in the real case, the induced distribution over the probability simplex is more heavily weighted towards the edges and corners.

We can see an example by considering the case with n=2. Instead of starting with a uniform distribution over the surface of the Bloch sphere, one starts with a uniform distribution over the unit circle in a two-dimensional real vector space. Let γ be the angle around this circle measured from some chosen axis (once a density matrix has been specified, we will take this axis to be along one of the eigenstates of the density matrix). Then, γ is initially uniformly distributed. The parameter analogous to $y_{1}$ of the preceding section is $y=\sin^{2}\gamma$. Note that *y* runs from 0 to 1 as γ runs from 0 to π/2. The initial probability density $\tau_{r}\left(y\right)$ is therefore obtained from

(21) $$\tau_{r}\left(y\right)dy=\left(2/\pi \right)d\gamma ,$$

which leads to

(22) $$\tau_{r}\left(y\right)=\frac{1}{\pi }\cdot \frac{1}{\sqrt{y(1-y)}}$$

(the subscript *r* represents “real”). This is in contrast to the function τ(y)=1 that would apply in the complex-amplitude case. We see that in the real case, $\tau_{r}\left(y\right)$ is largest around y=0 and y=1.

For *n* dimensions, we take as our parameters specifying a pure state (i) the first n-1 probabilities $y_{j}$ (j=1,…,n-1) of the outcomes of a certain orthogonal measurement (which we will choose to be the measurement along the eigenvectors of the given density matrix), and (ii) a set of discrete phase parameters (each of them taking the values ±1), which will always be independently and uniformly distributed and therefore suppressed in our expressions for the probability densities.

For the uniform distribution over the unit sphere in the *n*-dimensional real Hilbert space, one can show that the induced distribution over the parameters $\left(y_{1},\ldots ,y_{n-1}\right)$ is given by [26]

(23) $$\tau_{r}\left(y\right)=\frac{\Gamma (n/2)}{\pi^{n/2}}\cdot \frac{1}{\sqrt{y_{1}\cdots y_{n}}},$$

where $y_{n}=1-y_{1}-\cdots -y_{n-1}$. This probability density is normalized over the probability simplex, as in Equation (19):

(24) $$\int_{0}^{1}\int_{0}^{1-y_{1}}\cdots \int_{0}^{1-y_{1}-\cdots -y_{n-2}}\tau_{r}\left(y\right)dy_{n-1}\cdots dy_{2}dy_{1}=1.$$

The general expression for σ(x) given in Equation (11) remains valid in the real case, as do Equations (15)–(17) for the various factors in Equation (11). Again, the one difference is in $\tau_{r}\left(y\right)$, for which we now use Equation (23). By combining these ingredients, we arrive at our expression for the real-amplitude Scrooge ensemble:

(25) $$\sigma_{r}\left(x\right)=\frac{n\Gamma (n/2)}{\pi^{n/2}\sqrt{\lambda_{1}\cdots \lambda_{n}}\sqrt{x_{1}\cdots x_{n}}{\left(\frac{x_{1}}{\lambda_{1}}+\cdots +\frac{x_{n}}{\lambda_{n}}\right)}^{\frac{n}{2}+1}},$$

where, as before, the $\lambda_{j}$’s are the eigenvalues of the density matrix whose Scrooge distribution is being computed.

Though Equation (25) was derived as a distribution over the set of pure states in real-amplitude quantum theory, it reads as a probability distribution over the (n-1)-dimensional probability simplex for a classical random variable with *n* possible values. One can therefore at least imagine that there might be a classical scenario in which this distribution is natural. In the following section, we identify such a scenario.

## 4. Communicating with Dice

Ref. [26] imagined the following classical communication scenario. Alice is trying to convey to Bob the location of a point in an (n-1)-dimensional probability simplex. To do this, she constructs a weighted *n*-sided die that, for Bob, has the probabilities corresponding to the point that Alice is trying to convey. She then sends the die to Bob, who rolls the die many times in order to estimate the probabilities of the various possible outcomes. However, the information transmission is limited in that Bob is allowed only a fixed number of rolls—let us call this number *N* (perhaps the die automatically self-destructs after *N* rolls). So, Bob will always have an imperfect estimate of the probabilities that Alice is trying to convey. Alice and Bob are allowed to choose in advance a discrete set of points in the probability simplex—these are the points representing the set of signals Alice might try to send—and they choose this set of points, along with their *a priori* weights, so as to maximize the mutual information between the identity of the point being conveyed and the result of Bob’s rolls of the die. The main result of that paper was that in the limit of a large *N*, the optimal distribution of points in the probability simplex approximates the continuous distribution over the simplex expressed by the following probability density:

(26) $$\hat{\tau }\left(y\right)=\frac{\Gamma (n/2)}{\pi^{n/2}}\cdot \frac{1}{\sqrt{y_{1}\cdots y_{n}}},$$

where the $y_{j}$s are the probabilities (we use a hat in our labels of probability densities that arise in a classical context). This result is interesting because it is the same probability density as the one induced by the uniform distribution over the unit sphere in real Hilbert space (Equation (23) above). Thus, in a world based on real-amplitude quantum theory as opposed to the complex-amplitude theory, there is a sense in which one could say that nature optimizes the transfer of information.

That paper—and closely related papers [27,28]—deal only with the uniform distribution over the unit sphere, not with non-trivial Scrooge distributions. In the present section, we consider a modification of the above communication scenario, and in the next section, we show that this modified scheme yields the real-amplitude Scrooge distribution.

A natural way to generalize the above communication scheme is this: let the allowed number *N* of rolls of the die vary from one die to another (that is, some dice last longer than others before they self-destruct). Now, once *N* is allowed to vary, it makes sense to let *N* itself be another random variable that conveys information. We are thus led to consider the following scenario.

Alice is trying to convey to Bob an ordered *n*-tuple of non-negative real numbers $\left(M_{1},\ldots ,M_{n}\right)$ (Alice and Bob agree in advance on a specific set of such ordered *n*-tuples, any one of which Alice might try to convey). Let us refer to such an *n*-tuple as a “signal.” In order to convey her signal, Alice sends Bob an *n*-sided die that Bob then begins to roll over and over, keeping track of the number of times each outcome occurs. $N_{j}$ is the number of times that the outcome *j* occurs. At some point, the die self-destructs. Alice has constructed both the weighting of the die and the self-destruction mechanism so that the *average* value of $N_{j}$ is $M_{j}$.

However, both the rolling of the die and its duration are probabilistic, and Alice cannot completely control either the individual numbers $N_{j}$ or their sum. For any given signal $\left(M_{1},\ldots ,M_{n}\right)$, we assume that each $N_{j}$ is distributed independently according to a Poisson distribution with mean value $M_{j}$:

(27) $$P\left(N_{1},\ldots ,N_{n}|M_{1},\ldots ,M_{n}\right)=\prod_{j=1}^{n}e^{-M_{j}}\frac{M_{j}^{N_{j}}}{N_{j}!}.$$

This is equivalent to assuming that the *total* number *N* of rolls of the die is Poisson distributed with a mean value of $M=M_{1}+\cdots +M_{n}$ and that for a given total number of rolls, the numbers of occurrences of the individual outcomes are distributed according to a multinomial distribution with weights $M_{j}/M$. That is, we are assuming the usual statistics for rolling a die, together with a Poisson distribution for the total number of rolls (another model we could have used is to have Alice send Bob a radioactive sample that can decay in *n* ways and that Bob is allowed to observe with detectors for a fixed amount of time).

To make the problem interesting, and to keep Alice from being able to send Bob an arbitrarily large amount of information in a single die, limits are placed on the sizes of $M_{1},\ldots ,M_{n}$. This is done by imposing, for each *j*, an upper bound $M_{j}$ (script *M*) on the expectation value of the number of times the *j* outcome occurs. This expectation value is an average over all the possible signals that Alice might send.

We also need to say in what sense Alice and Bob are optimizing their communication. There are a number of reasonable options for doing this—e.g., we could say they maximize the mutual information, or minimize the probability of error for a fixed number of signals—but it is likely that many of these formulations will be essentially equivalent when the values of $M_{j}$ become very large. Here, we take a simple, informal approach. We say that, in order to make the various signals distinguishable from each other, Alice and Bob choose their *n*-tuples $\left(M_{1},\ldots ,M_{n}\right)$ so that neighboring signals, say $\left(M_{1},\ldots ,M_{n}\right)$ and $\left(M_{1}+\Delta M_{1},\ldots ,M_{n}+\Delta M_{n}\right)$, are at least a certain distance from each other, and we use the Fisher information metric to measure distance. Specifically, we require the Fisher information distance between the probability distributions $P(N_{1},\ldots ,N_{n}|M_{1},\ldots ,M_{n})$ and $P(N_{1},\ldots ,N_{n}|M_{1}+\Delta M_{1},\ldots ,M_{n}+\Delta M_{1})$ to be greater than or equal to a specified value $d_{min}$ (or, equivalently for small $\Delta M_{j}/M_{j}$, we require the Kullback–Leibler divergence to be at least $\left(1/2\right)d_{min}^{2}$). For the Poisson distribution and for small values of the ratios $\Delta M_{j}/M_{j}$, this condition works out to be

(28) $$\sum_{j=1}^{n}\frac{{\left(\Delta M_{j}\right)}^{2}}{M_{j}}\geq d_{min}^{2}.$$

For our purposes the exact value of $d_{min}$ is not important. We also assume that the various signals have equal *a priori* probabilities. This is a natural choice if one wants to convey as much information as possible. Under these assumptions, Alice and Bob’s aim is to maximize the number of distinct signals.

The analysis will be much simpler if we parameterize each die not by $\left(M_{1},\ldots ,M_{n}\right)$, but rather by the variables

(29) $$\alpha_{j}=\sqrt{M_{j}},\;j=1,\ldots ,n.$$

Then, for neighboring signals we can write

(30) $$\Delta \alpha_{j}=\frac{1}{2\sqrt{M_{j}}}\Delta M_{j},$$

so that the condition in Equation (28) becomes

(31) $$\sum_{j=1}^{n}{\left(\Delta \alpha_{j}\right)}^{2}\geq \frac{1}{4}\;d_{min}^{2}.$$

That is, in the space parameterized by $\vec{\alpha }=\left(\alpha_{1},\ldots ,\alpha_{n}\right)$, we want the points representing Alice’s signals to be evenly separated from each other. Thus Alice’s signals will be roughly uniformly distributed over some region of $\vec{\alpha }$-space—she wants to pack in as many signals as possible without exceeding the bounds $M_{j}$ on the expectation values of the $N_{j}$s. In what follows, we approximate this discrete but roughly uniform distribution of the values of $\vec{\alpha }$ by a continuous probability distribution. The probability density is zero outside the region where Alice’s possible signals lie; inside that region, it has a constant value of 1/V, where *V* is the volume of the region.

The communication problem then becomes a straightforward geometry problem—within the “positive” section of $\vec{\alpha }$-space (that is, the section in which each $\alpha_{j}$ is non-negative), the aim is to find the region R of largest volume that satisfies the constraints

(32) $$\frac{1}{V_{R}}\int_{R}\alpha_{j}^{2}\;d\vec{\alpha }=M_{j},\;j=1,\ldots ,n,$$

where $V_{R}$ is the volume of R. We maximize the volume because Alice’s signals have a fixed packing density within R; thus the larger the volume, the more signals Alice has at her disposal.

It is not hard to see that the solution to this geometry problem is to make region R the positive section of a certain ellipsoid centered at the origin. To see this, the conditions (32) can be written as

(33) $$\int_{R}\alpha_{j}^{2}\;d\vec{\alpha }=M_{j}\int_{R}d\vec{\alpha },\;j=1,\ldots ,n.\;$$

Now, let $\beta_{j}=\alpha_{j}/\sqrt{M_{j}}$. In terms of the $\beta_{j}$s, the above conditions become

(34) $$\int_{R^{\prime }}\beta_{j}^{2}\;d\vec{\beta }=\int_{R^{\prime }}d\vec{\beta },\;j=1,\ldots ,n,$$

where $R^{\prime }$ is the region of $\vec{\beta }$-space corresponding to the region R of $\vec{\alpha }$-space. In particular, the equation obtained by summing these *n* conditions must also be true:

(35) $$\int_{R^{\prime }}\beta^{2}\;d\vec{\beta }=n\int_{R^{\prime }}d\vec{\beta },$$

where $\beta^{2}=\beta_{1}^{2}+\cdots +\beta_{n}^{2}$. That is, the average squared distance from the origin over region $R^{\prime }$ must be equal to *n*. The maximum volume region $R^{\prime }$ satisfying this one condition is the positive section of a sphere, and one can work out that the radius of the sphere must be $\sqrt{n+2}$. Moreover, that region also satisfies all of the conditions (34). So, that same region is the maximum volume region that satisfies those conditions as well. Going back to the $\alpha_{j}$’s, we see that the maximum volume region satisfying the conditions (32) is the positive section of an ellipsoid, with semi-axis lengths

(36) $$\alpha_{j}^{max}=\sqrt{\left(n+2\right)M_{j}}.$$

Thus, the strategy that Alice and Bob adopt is to choose a set of closely packed signals with some minimum separation in $\vec{\alpha }$-space that occupies the positive section of an ellipsoid centered at the origin. Again, in this paper, we treat this discrete but roughly uniform distribution of signals as if it were actually uniform. This approximation becomes more and more reasonable as the values of the $M_{j}$s increase.

## 5. A Distribution over the Probability Simplex

So far, we have not made any connection between our communication problem and the real-amplitude Scrooge distribution. We do this now by seeing how the uniform distribution over the ellipsoid in $\vec{\alpha }$-space induces a certain probability distribution over the (n-1)-dimensional probability simplex for Alice’s *n*-sided die. We define this probability distribution as follows.

Let us imagine many rounds of communication from Alice to Bob: she has sent him many dice for which the expected numbers of occurrences of the various outcomes, $\left(M_{1},\ldots ,M_{n}\right)$, cover a representative range of values: the corresponding values of $\vec{\alpha }$ are distributed fairly uniformly over the region R in $\vec{\alpha }$-space. Bob has rolled each of these dice as many times as it can be rolled. Now consider a small region of the probability simplex, say the region S(x,Δx) for which the probability of the *j*th outcome lies between $x_{j}$ and $x_{j}+\Delta x_{j}$ for j=1,…,n-1. Some of the dice Alice has sent to Bob have probabilities lying in this region. The weight we want to attach to the region S(x,Δx) is, roughly speaking, the fraction of the total number of rolls that came from dice in this region. Note that for a die at location $\vec{\alpha }$, the expectation value of the number of times it will be rolled is $\alpha^{2}=\alpha_{1}^{2}+\cdots +\alpha_{n}^{2}$. So, we multiply the density of signals by the factor $\alpha^{2}$ to get the “density of rolls.” These considerations lead us to the following definition of the weight $\hat{\sigma }\left(x\right)dx_{1}\cdots dx_{n-1}$ that we assign to the infinitesimal region S(x,dx):

(37) $$\hat{\sigma }\left(x\right)dx_{1}\cdots dx_{n-1}=\frac{\int_{C(x,dx)}\alpha^{2}d\vec{\alpha }}{\int_{R}\alpha^{2}d\vec{\alpha }}.$$

Here, C(x,dx) is the cone (within the region R) representing dice for which the probabilities of the outcomes lie in S(x,dx):

(38) $$C\left(x,dx\right)=\left\{\vec{\alpha }\in R|x_{j}\leq \frac{\alpha_{j}^{2}}{\alpha^{2}}\leq x_{j}+dx_{j}\right\}.$$

Our use of the weighting factor $\alpha^{2}$ is reminiscent of the “adjustment” stage in the construction of the GAP measure in Refs. [5,6,7,8], and the integration over C(x,dx) is reminiscent of the projection stage of that same construction. We can express $\hat{\sigma }\left(x\right)$ more formally as

(39) $$\hat{\sigma }\left(x\right)=\frac{\int_{R}\left[\prod_{j=1}^{n-1}\delta \left(x_{j}-\frac{\alpha_{j}^{2}}{\alpha^{2}}\right)\right]\alpha^{2}d\vec{\alpha }}{\int_{R}\alpha^{2}d\vec{\alpha }},$$

where δ is the Dirac delta function.

It is not difficult to obtain an explicit expression for $\hat{\sigma }\left(x\right)$ starting with Equation (39). For example, in the integral appearing in the numerator of that equation, one can use the integration variables $s_{1},\ldots ,s_{n-1}$ and α, where $s_{j}=\alpha_{j}/\alpha$. Then, $d\vec{\alpha }$ becomes $\left(1/s_{n}\right)\alpha^{n-1}ds_{1}\ldots ds_{n-1}d\alpha$, and the integral becomes straightforward. Here, though, we take a different path to the same answer, starting with Equation (37). This latter approach turns out to be more parallel to our derivation of the Scrooge distribution in the quantum mechanical setting.

First, note that the numerator in Equation (37) can be written as

(40) $$\int_{C(x,dx)}\alpha^{2}d\vec{\alpha }=\frac{n}{n+2}\alpha_{max}^{2}\cdot (\mathrm{volume}\;\mathrm{of}\;C(x,dx)),$$

where $\alpha_{max}$ is the largest value of α over all points in R satisfying $\alpha_{j}^{2}/\alpha^{2}=x_{j}$ for j=1,…,n. We get Equation (40) by writing $d\vec{\alpha }$ as $k\alpha^{n-1}d\alpha$, with some constant *k*, for the purpose of integrating over the cone. We can find the value of $\alpha_{max}$ by finding the point of intersection between (i) the ellipsoid that defines the boundary of R, given by

(41) $$\frac{\alpha_{1}^{2}}{\left(n+2\right)M_{1}}+\cdots +\frac{\alpha_{n}^{2}}{\left(n+2\right)M_{n}}=1,$$

and (ii) the line parameterized by α and defined by the equations

(42) $$\alpha_{j}=\sqrt{x_{j}}\alpha ,\;j=1,\ldots ,n.$$

The value of α at this intersection point is

(43) $$\alpha_{max}=\sqrt{\frac{n+2}{\frac{x_{1}}{M_{1}}+\cdots +\frac{x_{n}}{M_{n}}}}.$$

We can therefore rewrite Equation (40) as

(44) $$\int_{C(x,dx)}\alpha^{2}d\vec{\alpha }=\frac{n}{\frac{x_{1}}{M_{1}}+\cdots +\frac{x_{n}}{M_{n}}}\cdot (\mathrm{volume}\;\mathrm{of}\;C(x,dx)).$$

Meanwhile, it follows from Equation (32) that the denominator in Equation (37) is

(45) $$\int_{R}\alpha^{2}d\vec{\alpha }=\left(M_{1}+\cdots +M_{n}\right)V_{R}.$$

Our next step is to compare $\hat{\sigma }\left(x\right)$ to the analogous distribution $\hat{\tau }\left(y\right)$ induced by the uniform distribution of the vector $\vec{\beta }$—the same $\vec{\beta }$ as in Section 4—over its domain $R^{\prime }$ (recall that $R^{\prime }$ is the positive section of a sphere):

(46) $$\hat{\tau }\left(y\right)dy_{1}\cdots dy_{n-1}=\frac{\int_{C^{\prime }\left(y,dy\right)}\beta^{2}d\vec{\beta }}{\int_{R^{\prime }}\beta^{2}d\vec{\beta }}.\;$$

Here, $C^{\prime }\left(y,dy\right)$ is the cone in $R^{\prime }$ for which $y_{j}\leq {\left(\beta_{j}/\beta \right)}^{2}\leq y_{j}+dy_{j}$. We can immediately write down an explicit expression for $\hat{\tau }\left(y\right)$. It is the same as the distribution (23) on the probability simplex induced by the uniform distribution over the unit sphere in the *n*-dimensional real Hilbert space—the extra radial dimension represented by β has no bearing on the distribution over the probability simplex. Thus,

(47) $$\hat{\tau }\left(y\right)=\frac{\Gamma (n/2)}{\pi^{n/2}}\cdot \frac{1}{\sqrt{y_{1}\cdots y_{n}}}.$$

The expression for $\hat{\sigma }\left(x\right)$ is determined by finding the factors by which the numerator and denominator in Equation (46) change when the sphere in $\vec{\beta }$-space is stretched into an ellipsoid in $\vec{\alpha }$-space. In this transformation (in which $\alpha_{j}=\beta_{j}\sqrt{M_{j}}$), the relation between *y* (in Equation (46)) and *x* (in Equation (37)) is given by y=g(x), where *g* takes the point $\left(\alpha_{1}^{2}/\alpha^{2},\ldots ,\alpha_{n-1}^{2}/\alpha^{2}\right)$ in the probability simplex to the point $\left(\beta_{1}^{2}/\beta^{2},\ldots ,\beta_{n-1}^{2}/\beta^{2}\right)$.

Essentially, any appearance of $M_{j}$ in our expression (37) for $\hat{\sigma }\left(x\right)dx_{1}\ldots dx_{n-1}$ becomes a 1 in Equation (46). Thus, according to Equation (44), when we transform from $\vec{\beta }$ to $\vec{\alpha }$, the numerator in Equation (46) is multiplied by

(48) $$\frac{\frac{n}{\frac{x_{1}}{M_{1}}+\cdots +\frac{x_{n}}{M_{n}}}\cdot (\mathrm{volume}\;\mathrm{of}\;C(x,dx))}{n\cdot (\mathrm{volume}\;\mathrm{of}\;C^{\prime }\left(y,dy\right))},$$

and according to Equation (45), in this same transformation, the denominator in Equation (46) is multiplied by

(49) $$\frac{\left(M_{1}+\cdots +M_{n}\right)V_{R}}{nV_{R^{\prime }}}.$$

For both the transitions $C^{\prime }\left(y,dy\right)\to C\left(x,dx\right)$ and $R^{\prime }\to R$, the volume increases by a factor of $\sqrt{M_{1}\cdots M_{n}}$. So, these volume factors cancel out. By inserting the other factors from Equations (48) and (49), it is found that

(50) $$\hat{\sigma }\left(x\right)=\hat{\tau }\left(y\right)J\left(y/x\right)\frac{n}{\left(\frac{x_{1}}{M_{1}}+\cdots +\frac{x_{n}}{M_{n}}\right)\left(M_{1}+\cdots +M_{n}\right)},$$

where J(y/x) is the Jacobian of *y* with respect to *x*.

Let us now write *y* explicitly in terms of *x*:

(51) $$y_{j}=\frac{\beta_{j}^{2}}{\beta^{2}}=\frac{\frac{\alpha_{j}^{2}}{M_{j}}}{\frac{\alpha_{1}^{2}}{M_{1}}+\cdots +\frac{\alpha_{n}^{2}}{M_{n}}}=\frac{\frac{x_{j}}{M_{j}}}{\frac{x_{1}}{M_{1}}+\cdots +\frac{x_{n}}{M_{n}}}.$$

From this, we can get the Jacobian (very much like the one in Equation (16)):

(52) $$J\left(y/x\right)=\frac{1}{M_{1}\cdots M_{n}}\cdot \frac{1}{{\left(\frac{x_{1}}{M_{1}}+\cdots +\frac{x_{n}}{M_{n}}\right)}^{n}}.$$

By inserting the results of Equations (51) and (52) into Equation (50), we arrive at

(53) $$\hat{\sigma }\left(x\right)=\frac{n\Gamma (n/2)}{\pi^{n/2}M\sqrt{M_{1}\cdots M_{n}}\sqrt{x_{1}\cdots x_{n}}{\left(\frac{x_{1}}{M_{1}}+\cdots +\frac{x_{n}}{M_{n}}\right)}^{\frac{n}{2}+1}},$$

where $M=M_{1}+\cdots +M_{n}$. This is essentially the same as the expression (25) obtained earlier as the real-amplitude Scrooge distribution. The agreement can be made more explicit by defining the ratios $\lambda_{j}=M_{j}/M$, in which case Equation (53) becomes exactly identical to Equation (25), with these $\lambda_{j}$s playing the role of the eigenvalues of the density matrix.

Note that in the above derivation, we see an analog of ρ distortion. The stretching of the sphere in $\vec{\beta }$-space into an ellipsoid in $\vec{\alpha }$-space is very much like ρ distortion, though in place of the notion of a density matrix, we have a uniform distribution within the sphere or ellipsoid.

It may seem that our communication set-up, in which Alice sends a die equipped with a probabilistic self-destruction mechanism, is rather artificial. However, the mathematics is actually fairly simple and natural. We are considering a set of Poisson-distributed random variables and are basically constructing a measure on the set of values of these variables based on distinguishability (this is the measure derived from the Fisher information metric). That measure then induces a measure on the probability simplex which agrees with the real-amplitude Scrooge distribution.

## 6. A Classical Interpretation of the Complex-Amplitude Scrooge Distribution

We now show how to modify the above classical communication scenario to arrive at the original, complex-amplitude Scrooge distribution.

Not surprisingly, we begin by doubling the number of sides of Alice’s dice. Let the outcomes be labeled $1_{a},1_{b},2_{a},2_{b},\ldots ,n_{a},n_{b}$. The communication scheme is exactly as it was in Section 4, except that instead of placing an upper bound on the expectation value of the number of times each individual outcome occurs, the $j_{a}$ and $j_{b}$ outcomes are grouped together and an upper bound $M_{j}$ is placed on the expectation value of the total number of times the two *j* outcomes occur. This is done for each j=1,…,n. Again, Alice and Bob are asked to maximize the number of distinguishable signals under this constraint, where “distinguishable” again means having a Fisher-distance separation of at least $d_{min}$.

As before, it is easiest to view the problem in $\vec{\alpha }$-space; let us label the variables in the space $\alpha_{ja}$ and $\alpha_{jb}$. We now look for the maximum-volume region R of the positive section of $\vec{\alpha }$-space satisfying the constraints

(54) $$\frac{1}{V_{R}}\int_{R}\left(\alpha_{ja}^{2}+\alpha_{jb}^{2}\right)d\vec{\alpha }=M_{j},\;j=1,\ldots ,n.$$

In terms of the variables $\beta_{ja}=\alpha_{ja}/\sqrt{M_{j}}$ and $\beta_{jb}=\alpha_{jb}/\sqrt{M_{j}}$, the constraints become

(55) $$\frac{1}{V_{R^{\prime }}}\int_{R^{\prime }}\left(\beta_{ja}^{2}+\beta_{jb}^{2}\right)d\vec{\beta }=1,\;j=1,\ldots ,n,$$

where $R^{\prime }$ is the region in $\vec{\beta }$-space corresponding to R. Upon summing these *n* constraints, the equation

(56) $$\frac{1}{V_{R^{\prime }}}\int_{R^{\prime }}\beta^{2}d\vec{\beta }=n$$

is obtained, where $\beta^{2}=\sum_{j=1}^{n}\left(\beta_{ja}^{2}+\beta_{jb}^{2}\right)$. Maximizing the volume under this constraint again gives a sphere in $\vec{\beta }$-space, which becomes an ellipsoid in $\vec{\alpha }$-space (restricted to the positive section).

Continuing as before, one finds that the induced probability distribution over the (2n-1)-dimensional probability simplex associated with a 2n-sided die is the analog of Equation (53), the *n* values $M_{1},\ldots ,M_{n}$ now being replaced by the 2n values $M_{1}/2,M_{1}/2,\ldots ,M_{n}/2,M_{n}/2$.

(57) $${\hat{\sigma }}_{ab}\left(x\right)=\frac{n\Gamma (n)}{\pi^{n}\lambda_{1}\cdots \lambda_{n}\sqrt{x_{1a}x_{1b}\cdots x_{na}x_{nb}}{\left(\frac{x_{1a}+x_{1b}}{\lambda_{1}}+\cdots +\frac{x_{na}+x_{nb}}{\lambda_{n}}\right)}^{n+1}},$$

where $\lambda_{j}=M_{j}/M$. Here, $x_{ja}$ and $x_{jb}$ are the probabilities of the outcomes $j_{a}$ and $j_{b}$, and x refers to the point $\left(x_{1a},x_{1b},\ldots ,x_{(n-1)a},x_{(n-1)b},x_{na}\right)$ in the (2n-1)-dimensional probability simplex (the value of $x_{nb}$ is determined by the requirement that the probabilities sum to unity).

Finally, a distribution over the (n-1)-dimensional probability simplex is obtained by ignoring the difference between the outcomes $j_{a}$ and $j_{b}$. We can imagine an observer who, unlike Alice and Bob, cannot see the *a* and *b*. For this “ab-blind” observer, the distribution of Equation (57) looks like the following distribution over the (n-1)-dimensional probability simplex:

(58) $$\hat{\sigma }\left(x\right)=\int \prod_{j=1}^{n-1}\delta \left[x_{j}-\left(x_{ja}+x_{jb}\right)\right]{\hat{\sigma }}_{ab}\left(x\right)dx_{1a}dx_{1b}\cdots dx_{na}.$$

Here, δ is the Dirac delta function and the integral is over the (2n-1)-dimensional probability simplex.

The integral in Equation (58) is straightforward, and it can be found that

(59) $$\hat{\sigma }\left(x\right)=\frac{n!}{\lambda_{1}\cdots \lambda_{n}}\cdot \frac{1}{{\left(\frac{x_{1}}{\lambda_{1}}+\cdots +\frac{x_{n}}{\lambda_{n}}\right)}^{n+1}}.$$

This is the same as the original Scrooge distribution of Equation (18). The role of the eigenvalues of the density matrix is now played by the set of values $\lambda_{j}=M_{j}/\left(M_{1}+\cdots +M_{n}\right)$, where, again, $M_{j}$ is the maximum allowed expectation value of the number of times that the outcomes $j_{a}$ and $j_{b}$ occur.

## 7. Discussion

In this paper we have shown how the real-amplitude version of the Scrooge distribution emerges naturally from a classical communication scenario in which information is transmitted via the values of several random variables $N_{j}$. Essentially, the real-amplitude Scrooge distribution, regarded as a probability distribution over the probability simplex, is derived from an underlying distribution based on distinguishability. Our analysis includes a transformation that plays something like the role of a ρ distortion: in place of a density matrix, what is distorted is a distribution over the space of potential signals.

In order to get the original complex-amplitude Scrooge distribution for dimension *n*, we needed to consider a case with twice as many random variables, grouped into pairs, and then we imagined an observer for whom only the *sum* of the variables within each pair was observable.

The reader will probably have noticed that the role played by the concept of *information* in our classical communication problem seems to be exactly the opposite of the role it plays in the quantum origin of the Scrooge distribution. In quantum theory, the Scrooge distribution is the distribution over pure states that, upon measurement, provides an observer with the *least* possible amount of information. In contrast, in our classical communication scenario, the Scrooge distribution emerges from a requirement that Alice convey as much information as possible to Bob. What is common to both cases is that the information-based criterion favors a distribution that is highly *spread out* over the probability simplex. In the quantum case, a distribution spread out over many non-orthogonal states tends to make it difficult for an observer to gain information about the state. In the classical case, Alice and Bob want to spread their signals as widely as possible over the space of possibilities in order to maximize the number of distinguishable signals. Thus, though the two scenarios are quite different, their extremization criteria have similar effects.

An intriguing aspect of our classical scenario is that the probability simplex is not itself taken as the domain in which the problem is formulated. Instead, the problem is formulated in terms of the number of times each outcome occurs. The distribution over the probability simplex is a secondary concept, being derived from a more fundamental distribution over the space of the numbers of occurrences of the outcomes. That is, the $M_{j}$ values are more fundamental in the problem than the probabilities of the outcomes, which are defined in terms of the $M_{j}$s by the equation $x_{j}=M_{j}/M$. In this specific respect, then, the effort to find a classical interpretation of the Scrooge distribution seems to lead us away from the models studied in Refs. [26,28], in which the set of frequencies of occurrence of the measurement outcomes was the only source of information considered.

It is interesting to ask whether this feature of our scenario is necessary in order to get the Scrooge distribution classically. To address this question, in Appendix A we consider another classical communication problem, in which we impose a separate restriction for each outcome as in Section 4, but now with Alice’s signals consisting purely of probabilities (which are estimated by Bob through the observed frequencies of occurrence). For simplicity, we restrict our attention to the most basic case, in which there are only two possible outcomes—so Alice’s die is now a coin to be tossed—and we are aiming just for the real-amplitude Scrooge distribution as opposed to the complex-amplitude version. We find that the resulting probability distribution over the probability simplex is *not* of the same form as the real-amplitude Scrooge distribution. This result can be taken as one bit of evidence that it is indeed necessary to go beyond the probability simplex and to work in a space of one additional dimension in order to obtain the Scrooge distribution classically. In this connection, it is worth noting that something very similar has been seen in research on *subentropy*—certain simple relations between subentropy and the Shannon entropy can be obtained only by lifting the normalization restriction that defines the probability simplex and working in the larger space of unnormalized *n*-tuples [21,23].

Finally, one might wonder about the potential significance of our need to invoke an “ab-blind” observer in order to obtain the complex-amplitude Scrooge distribution. It is well known that the number of independent parameters required to specify a pure quantum state (of a system with a finite-dimensional Hilbert space) is exactly twice the number of independent probabilities associated with a complete orthogonal measurement on the system. Here, we are seeing another manifestation of this factor of two: the classical measurement outcomes, corresponding to the sides of a rolled die, have to be grouped into pairs, and we need to imagine an observer incapable of distinguishing between the elements of any pair. In our actual quantum world, one can reasonably ask whether there is any interesting sense in which we ourselves are “ab-blind.” This question, though, lies well beyond the scope of the present paper.

## Appendix A. Communicating through Probabilities

Here, we consider a classical communication problem based directly on probabilities, as opposed to being based on the number of times each outcome occurs. We restrict our attention to the case of two outcomes, which we imagine as “heads” and “tails” for a tossed coin. The question is whether the real-amplitude Scrooge distribution for n=2 can be obtained in this way.

Alice is trying to convey to Bob the identity of a point in the one-dimensional probability simplex (not the two-dimensional space with axes labeled “number of heads” and “number of tails”). The “simplex” in this case is just a line segment, and the points of the simplex are labeled by the probability *x* of heads occurring (the probability of tails occurring is 1-x). Alice conveys her signal by sending Bob a coin with weights (x,1-x). Bob tosses the coin in order to estimate the value of *x*, but he is allowed to toss it only *N* times, at which point the coin will self-destruct. Alice chooses a set of points in the probability simplex in advance that will serve as her potential signals, and she provides Bob with the list of these points. Alice also chooses a function N(x) that determines how many times Bob will be able to toss the coin if the coin’s weights are (x,1-x). However, Bob does not know the function N(x) and is not allowed to use the observed total number of tosses in his estimation of the value of *x*. He can use only the frequencies of occurrence of heads and tails.

We limit the amount of information that Alice can convey per coin by specifying the values of two quantities: (i) the expectation value N of the total number of tosses, and (ii) the expectation value $N_{H}$ of the number of heads. If we let ρ(x)dx be the number of signals lying between the values *x* and x+dx, we can write these two restrictions as follows:

(A1) $$\int_{0}^{1}N\left(x\right)\rho \left(x\right)dx=N\int_{0}^{1}\rho \left(x\right)dx.$$

(A2) $$\int_{0}^{1}xN\left(x\right)\rho \left(x\right)dx=N_{H}\int_{0}^{1}\rho \left(x\right)dx.$$

As before, we insist that Alice choose the signal values so that neighboring signals have a certain minimum degree of distinguishability as quantified by the Fisher information metric. For the binomial distributions we are dealing with here, this condition works out to be

(A3) $$\Delta x=\sqrt{\frac{x(1-x)}{N(x)}}\;d_{min},$$

where Δx is the separation between successive signals. The density ρ(x) of signals is therefore

(A4) $$\rho \left(x\right)=\frac{1}{\Delta x}=\sqrt{\frac{N(x)}{x(1-x)}}\;\frac{1}{d_{min}}.$$

Alice wants to maximize the number of distinct signals. So, in choosing the function N(x), she needs to solve the following optimization problem: maximize the quantity (from Equation (A4))

(A5) $$\int_{0}^{1}\sqrt{\frac{N(x)}{x(1-x)}}dx,$$

while satisfying the following two constraints (which come from Equations (A1) and (A2), combined with Equation (A4))

(A6) $$\int_{0}^{1}\frac{N{\left(x\right)}^{3/2}-NN{\left(x\right)}^{1/2}}{\sqrt{x(1-x)}}dx=0.$$

(A7) $$\int_{0}^{1}\frac{xN{\left(x\right)}^{3/2}-N_{H}N{\left(x\right)}^{1/2}}{\sqrt{x(1-x)}}dx=0.$$

This problem can be solved by the calculus of variations, and it can be found that Alice should choose N(x) to be of the form

(A8) $$N\left(x\right)\propto \frac{1}{\frac{x}{\lambda }+\frac{1-x}{1-\lambda }}.$$

Here, λ is a real number between zero and one, fixed by the requirement that the overall probability of heads must equal $N_{H}/N$ (we could have written the result in other ways; we use λ only to facilitate our later comparison with the Scrooge distribution). Once the value of λ is set, the constant factor multiplying the right-hand side is fixed by Equation (A6).

We now use this result to generate the probability distribution $\hat{\sigma }\left(x\right)$ over the probability simplex. We define it as follows: in many rounds of communication, we want $\hat{\sigma }\left(x\right)dx$ to approximate the fraction of the total number of tosses that come from a coin whose probability of heads is between *x* and x+dx. More precisely, we define $\hat{\sigma }\left(x\right)$ to be proportional to N(x)ρ(x), with the proportionality constant set by the normalization condition $\int_{0}^{1}\hat{\sigma }\left(x\right)dx=1$ (we have multiplied ρ(x) by N(x) to turn the density of signals into the density of tosses). By substituting for N(x) and ρ(x) in accordance with Equations (A4) and (A8), we arrive at

(A9) $$\hat{\sigma }\left(x\right)=\frac{A}{\sqrt{x(1-x)}}\cdot \frac{1}{{\left(\frac{x}{\lambda }+\frac{1-x}{1-\lambda }\right)}^{3/2}},$$

where *A* is the normalization constant. Comparing this form with that of Equation (25), we see that this alternative problem does not lead us to the real-amplitude Scrooge distribution—the exponent appearing in the denominator is 3/2 instead of 2. Moreover, λ and 1-λ have no obvious meaning in this problem, whereas in the problem considered in Section 4 and Section 5, the $\lambda_{j}$s can be interpreted directly in terms of the imposed bounds $M_{j}$ on the expectation values of the number of times that the various outcomes occur.
